# Insects, Plants, and Microorganisms from Dry Lands as Novel Sources of Proteins and Peptides for Human Consumption

**DOI:** 10.3390/foods12234284

**Published:** 2023-11-27

**Authors:** Nathiely Ramírez-Guzmán, Cristian Torres-León, David Aguillón-Gutiérrez, Jorge Alejandro Aguirre-Joya

**Affiliations:** 1 Center for Interdisciplinary Studies and Research (CEII-UAdeC), Universidad Autónoma de Coahuila, Saltillo 25280, Mexico; nathiely.ramirez@uadec.edu.mx; 2 Reaserch Center and Ethnobiological Garden (CIJE), Universidad Autónoma de Coahuila, Viesca 27480, Mexico; ctorresleon@uadec.edu.mx (C.T.-L.); david_aguillon@uadec.edu.mx (D.A.-G.)

**Keywords:** proteins, novel sources, dry lands, insects, plants, microorganisms

## Abstract

Protein malnutrition is present in developing countries but also in developed ones due to actual eating habits involving insufficient protein intake. In addition to this, it is estimated by the Food and Agricultural Organization of the United Nations that the world’s population will increase to 9.1 billion people in less than 30 years. This poses a significant challenge in terms of nourishing the population. Different strategies have been proposed to address this challenge, including exploring novel protein sources such as plants. For instance, Prosopis alba pods have an 85.5% protein content. Other examples are microorganisms, such as *Halobacillus adaensis* which produces 571 U/mL of protease, and insects such as those belonging to the Orthoptera order, like grasshoppers, which have a protein content of 65.96%. These sources have been found in dry lands and are being explored to address this challenge.

## 1. Introduction

According to the Food and Agriculture Organization of the United Nations (FAO), a world population of 9.1 billion people is expected by 2050, and food production needs to increase by 70% to feed the world [1]. Despite the actual global production level of food, protein malnutrition is still present in developing and developed countries due to actual eating habits involving insufficient protein levels in the diet [2].

Proteins are fundamental natural substances with bioactive activities, found in a wide range of species; in addition, peptides are protein fragments that provide the nutrients needed for human growth and development. Typically, these peptides are constituted by the union of 2 to 20 amino acids linked to each other via peptide bonds [3]. 

Bioactive peptides are defined as those that promote beneficial health effects, so food-derived bioactive peptides can be found naturally in food sources and have beneficial health effects.

The main source of protein for human consumption is the meat industry. Nevertheless, meat production is responsible for 54% of greenhouse gases generated by agricultural activities, with a predicted increase to 59% by 2030 [4]. Another worrying aspect of the dependency on meat protein is the associated water footprint. 

Therefore, there is an urgent need to develop a new strategy to meet the huge demand for proteins of a growing worldwide population, while also considering consumers’ sensory and nutritional needs and a lower negative environmental impact. 

This paper points to novel protein sources from dry lands for human consumption from different origins, such as insects, microorganisms, and plants. 

## 2. Microorganisms Isolated from Arid Areas of Interest for the Production of Peptides and/or Proteins for Human Consumption

Arid and semiarid zones are defined as geographic regions characterized by extreme and complicated climates where extreme dryness predominates; these regions experience prolonged periods of drought, sporadic rain with low average amounts, and generally high temperatures in the day and low temperatures at night [5,6]. This causes the vegetation in these regions to be reduced or almost absent; this affects the biodiversity of these areas since it influences the behavior and processes of the species that live there as they adapt to live in such conditions [6]. Arid and semiarid zones are of great importance for conserving regional biological diversity and maintaining the ecological processes that occur there [7].

Generally, since this is the visible part of any ecosystem, flora and fauna are important factors (in addition to atmospheric conditions) when classifying and naming the different geographical areas on Earth [8]. However, other elements present in each of the ecosystems are microorganisms since they vitally participate in ecosystems and constantly interact with animals, plants, and human beings [5].

Microorganisms are the most primitive and numerous organisms that exist on Earth; they colonize every environment: soil, water, and air. It is estimated that there are about a nonillion microorganisms; however, only 1% are known, partly because many areas of the Earth remain unexplored, where a huge diversity of microorganisms with interesting and unique characteristics may exist [9]. Microorganisms are classified in many ways, including their ability to grow at different temperatures. In contrast, mesophylls grow at moderate temperatures, and this helps them to be cultivated and therefore be better known and studied. Some organisms, however, live in extreme conditions compared to those considered normal, even in locations where it might be thought that it is not possible for there to be life [10,11]. Extremophiles represent microorganisms that can live in nonphysiological conditions, including pressure, salinity, pH value, heavy metals, radiation, and temperature [12,13].

A subtype of these are the thermophiles that endure extreme conditions of relatively high temperature, above 45 °C, which is why it is normal to find this type of microorganism in arid areas. Each environmental condition causes the organisms to acquire a variety of adaptation techniques, which makes them stable in a unique way in that specific environment, including alterations to their metabolism and the ability to produce substances that allow them to survive, which are generally of high biotechnological and industrial interest, such as in the food industry [5,14,15].

In recent years, interest in the study and development of functional and nutraceutical foods has increased greatly, given the great economic impact of the commercialization of this type of food and the products that contain them [16]. The constant changes in society’s eating habits in the search for a healthier life have focused on diets high in protein and bioactive peptides for their nutritional value and biological properties [17]. The most common sources of obtaining these molecules are animal and plant sources. However, in recent years, an important niche has been discovered in their production from or through microbial processes. There are two options: that the microorganism produces these molecules or that it generates a compound such as the enzymes that are secreted from its metabolism; that it helps to produce or synthesize some protein or peptide from another source [18,19].

To obtain bioactive peptides, they must be released from the parental protein in which they are encrypted and inactive. There are several processes for obtaining bioactive peptides: chemical synthesis, chemical hydrolysis, cooking, maturation, digestion, and recombinant DNA technology. However, biotechnological methods such as microbial fermentation and enzymatic hydrolysis are the most commonly used.

In October 2023, the online database Web of Science Core Collection (WoSCC) was accessed with the following search string: “Insects” OR “plants” OR “microorganisms”. The search string was applied to the title, abstract, and author keywords fields of 1,037,396 indexed publications. After adding an additional filter, “protein”, 143,514 publications were returned. After adding an “arid zone” filter, the search returned 15 results that were used for our analysis.

## 3. Microorganisms from Arid Zones as a Source of Protein for Human Consumption

In general, microbial fermentations or enzymatic hydrolysis to obtain bioactive peptides and proteins are carried out using strains isolated from dairy products, with the predominance of bacillus species, especially lactic acid bacteria belonging to the genus *Lactobacillus*, which are prominent microbial groups [20,21,22]. The reason for this type of bacteria’s efficient production of peptides may be related to their amino acid auxotrophy [23]. Therefore, fewer works have been carried out with microorganisms isolated from other sources.

An example of obtaining bioactive peptides from microbial fermentation is presented in the study by Gulyamova et al [24]. They detected that bioactive peptides inhibited angiotensin-converting enzyme I, showing the potential to reduce blood pressure. They used dochi, a popular condiment in Chinese gastronomy that consists of the fermentation of soybeans by *Aspergillus egypticus.* The study evaluated different conditions, such as duration and secondary fermentation. *Aspergillus egypticus* was first described in 1972. It was isolated from sandy soils in Egypt and used in different studies and used to inhibit pancreatic α-amylase activity [24,25].

*Bifidobacterium* is a genus that belongs to the lactic acid producers’ bacteria was reported to have the capacity to produce many bioactive metabolites, including bioactive peptides [26].

In particular, *Bifidobacterium animalis*, *Bifidobacterium longum*, and *Bifidobacterium pseudolongum* have triggered scientific interest due to their capability to produce or synthesize novel peptides. Scientific reports describe the production of 25 novel peptides [27].

Delgado-García et al [28] produced bioactive peptides from fish waste using proteases from halophilic microorganisms isolated from two localities classified as arid zones in South America, the Uyuni desert in Bolivia and Cuatro Ciénegas Coahuila in México, which are characterized by their sparse vegetation and specific climates. The microorganisms isolated in Uyuni, Bolivia, were identified as strains of *Halobacterium* sp. In the Cuatro Ciénegas region, the isolates were mainly bacteria of the *Bacillaceae* family, which are the most abundant bacteria in natural environments and include the genera of *Alkalibacillus* sp., *Marinococcus* sp., and *Halobacillus* sp. These genera presented a greater capacity to produce hydrolytic enzymes capable of degrading fish waste to produce bioactive peptides of food interest.

Delgado-García et al [29] isolated bacteria from the same region in the Coahuilense semi-desert in four different locations, including Cuatro Ciénegas. These authors reported the metabolic capacity of eight strains, *Salinicoccus roseus* (EC-01), *Halobacillus* sp. (AS-04), *Oceanobacillus* sp. (ES1-03), *Halobacillus trueperi* (CT2-03), *Bacillus pumilus* (CP-01), *Bacillus subtilis* (AS-09), *Bacillus atrophaeus* (PN-01), and *Bacillus atrophaeus* (SY-01), to produce enzymes for the degradation of starch, pectin, cellulose, and xylan. The production of enzymes such as amylases, pectinases, and xylanases is of interest in the human food industry since these enzymes intervene in the process of making food products, such as in the fruit-processing industry for clarification of juices and wines, in baking to improve the quality of the bread and decrease the viscosity of pasta, and in the brewing industry, among others [17,30,31,32].

Different enzymes such as hydrolases and proteases in the food industry are also important since these enzymes have been reported for various purposes such as producing dairy products, bakery, and clarifying xanthan gum [20,21,33,34]. Table 1 summarizes a list of microorganisms with the metabolic capacity necessary for producing these enzymes and the different arid zones from which they were obtained.

In extreme environments such as arid zones, we can find organisms distinguishable by their unique biochemistry and extraordinary physiological capacities resulting from adaptations to special environmental conditions. Their adaptations and particular characteristics can be useful for their application in numerous fields, such as food, due to their production of peptides and proteins. Despite this potential, they have not been widely studied [39,40,41].

## 4. Plants from Arid Zones as a Source of Protein for Human Consumption

Proteins are essential macronutrients in human nutrition. Plant proteins are made up of twenty-two essential amino acids [42]. Integrating proteins from diverse plant sources can supply adequate essential amino acids to fulfill human nutritional needs [43]. 

The search for new protein sources is a global priority, and plants grown in arid areas are a sustainable alternative to current sources. The great biological diversity of arid zones is a potential source of natural resources. Plants that grow in arid regions have some advantages; these plants are adapted to adverse growing conditions, require less water, and allow the use of infertile land [44]. Additionally, these resources can be used sustainably to improve rural populations’ quality of life [44]. Plant proteins will be essential when animal-derived proteins fail to satisfy the requirements of the global population [43]. It is increasingly important to find new crop plants or genotypes of crops that have adaptation strategies to water shortages in extremely arid conditions [45]. The range of vegetation in arid regions consists of shrub species, abundant cacti, and grasses [46].

Table 2 shows the main plants of arid regions that have been investigated as a source of protein with functional properties. Legumes are among the main plants with the potential for use in protein production. According to Singh and Abhilash [47], legumes are important crops for food and nutritional security, and legumes are second to cereals as a source of human nutrition. Underutilized legumes contain adequate quantities of essential amino acids compared to other crops [48].

*Lablab purpureus* (L.) Sweet in the Fabaceae family is locally named kashrangeeg in Nubia, Egypt [45]. The plant is used as a forage for grazing cattle, sheep, goats, and pigs. This plant is a legume of indeterminate growth, either annual or biennial, as determined by environmental conditions. Despite having so many benefits, this plant has been underutilized. Currently, there are only reports of cultivation in sub-tropical areas of Africa, Central and South America, the West Indies, Southeast Asia, and Indonesia (the crop can be used in rotation with cereals to add fixed N to the soil) [48]. According to Table 2, the protein content of *Lablab purpureus* (L.) is between 15% and 87.8%. Furthermore, Ahmed et al. [45] showed that *L. purpureus* is a good source of protein, concentrated in its seeds and pods, and can be used in many forms, such as the unripe pods and mature green seeds, with the haulm, leaves, dry basis, and straw used as livestock feed [49]. Additionally, this plant has the capacity to tolerate stress generated by drought and salinity [48]. It can be used as a food and feed legume as herds readily consume it in desert environments [45]. *L. purpureus* has a wide spectrum of therapeutic potential, showing cytostatic potential and anticancer activities [48]. This plant has other techno-functional properties, such as foaming, emulsification, and gelation. The performance of this crop under drought stress offers comparatively cost-effective nutrition [48].

On the American continent, two species of plants from arid zones have been studied as a source of protein. *Opuntia* spp. and *Prosopis* spp. have a low water requirement and excellent adaptation to semiarid regions [46]. 

*Opuntia* spp. contains between 7.7% and 13.8% protein (Table 2). *Opuntia ficus-indica* (prickly pears, nopal) belong to the *Cactaceae* family and grow in arid parts of the world [57]. Nopal is used in food and as forage. Nopal has traditionally been used in Mexico since pre-Columbian times by the Aztecs in culinary preparations [58]. The young stems of *Opuntia* spp., known as cladodes, are widely consumed in Mexico [50].

The FAO considers Prosopis species as multipurpose trees and shrubs, and their fruit constitutes a food source for humans and animals [55]. The flour obtained through grinding the pods of *Prosopis* spp. is rich in protein. This species has a protein content between 9.5% and 85.5% (Table 2), with valine as the only limiting amino acid [59]. Cattaneo et al. [55] suggested that *Prosopis* spp. flour protein isolate could be a new alternative in the formulation of foods for humans. Bigne et al. [60] mixed mesquite flour (150–350 g/kg) with wheat flour (650–850 g/kg) to obtain composite sweetbreads. Sensory analysis revealed a remarkable degree of acceptability for these mixed breads, particularly at the 250 g/kg replacement level.

The *Calligonum* genus (North African Sahara) is known for feeding purposes [61]. Bannour et al. [56] reported that *Calligonum azel* Maire flowers are protein-rich (Table 2). The authors concluded that this plant can be used as a food ingredient. 

The main disadvantage of arid-zone plants is the presence of anti-nutritional factors such as non-protein amino acids [62] and tannins, phytate (18.9 mg/g), and trypsin inhibitors (0.15 TIU/mg) [48,63]. Plants such as legumes and tree seeds such as the genera *proposis* spp. contain anti-nutritional factors that inhibit the activity of proteases in the gastrointestinal tract [46]. Although specific tannins like gallotannins can be functional, they generally have adverse effects when bound to proteins [64]. Before using these seeds as food, some treatments are needed to reduce their anti-nutritional factors (trypsin inhibitor and phytate) [63]. These anti-nutritional factors can be eliminated using cooking methods. Torres et al. [65]) reported the extraction of tannins by cooking and soaking mango seed (a by-product with a high tannins content of 0.19–0.44%). These methods are commonly used in flour production and can also be used to eliminate anti-nutritional factors in plants from arid zones [17]. Anti-nutritional compounds of mesquite pods can also be removed by roasting the pod at 150 °C for 45 min [46]. During protein isolation, a series of treatments or steps reduce the anti-nutritional factors to an undetectable level [66]. Garg et al. [53] reported that protein isolation from *Prosopis cineraria* (evaluating extraction factors using the response surface methodology) generates a 95% reduction in tannins. Mohan and Mellem [49] recently reported high digestibility values with protein efficiency ratios for isolates greater than 2 (2.61–6.66). According to the authors, the high digestibility results may suggest lower contents of anti-nutritional factors. 

Environmental factors affect plant development. Although anti-nutritional factors can be negative, the molecules generated in these climatic conditions also produce interesting functional properties in plant molecules. In these plants, heat stress decreases the leaf area and leads to the accumulation of reactive oxygen species (ROS); this makes the plants develop bioactive molecules that reduce the damaging effects of ROS. Table 2 shows the main functional properties reported in the plant proteins in arid zones. Due to the importance of desert plants and their important role in sustainable development, further research is needed to investigate the potential of other plants in arid zones as a source of protein. Their functional properties must also be investigated.

## 5. Insects from Arid Zones as a Source of Proteins and Peptides for Human Consumption

Insects are a food source for many cultures around the world. This means there are many specific people located in different countries that consider the use of insects as food as habitual aspect, particularly in sub-tropical and tropical regions in Asia, Africa, and Latin America [67]. Insects can be consumed at different stages of their development and be prepared in different ways, thus forming an important part of gastronomy in various places. Although there are cosmopolitan insects, the species of edible insects largely depends on the type of ecosystem in which a human population is found and the season.

Generally, edible insects are easy to identify and capture, exist in great numbers, and are present for a good part of the year. There are more than 2000 species of edible insects around the world. In addition to providing an important source of protein, they need proportionally less food and space than livestock to generate 1 kg of protein, and they minimize the contamination generated by food production in traditional livestock [68].

Edible insects are part of the traditional eating habits of Mexico, in particular. Their preparation and consumption have remained almost identical for centuries, and are sufficiently widespread to generate economic income, which is an important developmental consideration [69].

In addition, Mexico is the most entomophagous country on the planet, with 549 species of edible insects [70,71]. Although the consumption of insects and other arthropods in Mexico is more widespread in central and southern areas, this practice also occurs in the north; for example, in Durango, scorpions are consumed, even as a gourmet dish.

The production of edible insects in arid areas can largely solve the problem of food security, offering a highly nutritious and relatively inexpensive option, especially in those areas where water resources are scarce and, consequently, livestock and agriculture are no longer extensively viable [72].

Other productive alternatives in Mexico’s arid and semiarid zones are made up of some species of edible insects, such as wild bees and the honey ant, which are used mainly for self-consumption. Other species of insects have great economic potential because their consumption is very popular in the country’s central area; these species are the red and white maguey worm and escamoles, since they have a delicate flavor and high nutritional quality (40% protein) [73].

It is well known that the nutritional value of insects is high; for example, the protein content of edible insects ranges from 28% to 81%, expressed on a dry basis. Most species have 55% to 65% of good-quality protein; that is, half to almost three-quarters of their body is made up of proteins. Their digestibility, that is, their use, ranges from 75% to 98%, which implies that almost all of it is used.

Chemical ratings reach 96% (protein quality) in insects, only surpassed by those of egg and milk. The most edible insect orders are Hymenoptera, Orthoptera, Hemiptera, and Coleoptera, which have a protein content that ranges from 9.45% to 77.13% [74]. It is also known that in insects the protein content is lower than that of fat; on average, there is twice as much protein as fat, although in some cases, the protein can be up to seven times more. The insects that contain the highest amount of protein belong to the Orthoptera order (65.96%); on the contrary, those of the Coleoptera order contain the least (44.03%). Therefore, half of the dry weight in most insects is protein [75]. Some edible insect larvae in Mexico have a protein content ranging from 45.25% to 60.75%, all containing eight essential amino acids [72]. In conclusion, edible insects could alleviate poverty and malnutrition in arid and semiarid areas of the planet, offering an economically, environmentally, and socioculturally viable alternative to meat production.

An important aspect that diverse authors have studied in recent years is the rejection by consumers of the consumption of insects as whole products or as an ingredient (powdered or processed). Food neophobia is defined as a negative willingness to eat insects or food products with insects as ingredients. 

Recent studies indicate that people with positive experiences with insects as food present less disgust and increased willingness to taste food made with insects as an ingredient or eat a whole insect directly. Also, they highlight the importance of promoting entomophagy through cooking programs or tasting sessions to reduce disgust, so that people can accept insects as food [76].

## 6. Knowledge Summary and Future Directions

There has been increasing interest in studying and developing functional and nutraceutical foods. Also, novel sources of proteins or bioactive peptides are a central theme here due to different factors such as their economic, environmental, agricultural, and feed capacities. The meat industry is the main protein source for human consumption. However, this industry is responsible for a high amount of greenhouse gas production, specifically 54% of the total for agricultural activities, not to mention the water footprint. The present article describes interesting novel protein and peptide sources, like microorganisms, plants, and insects, from dry lands for human consumption with promising capacities. 

The growing need for proteins and peptides, coupled with the expected increase in humankind to 9.1 billion people by 2050 and actual protein production problems, mean it is necessary to explore novel protein and peptide sources for human consumption. Novel sources from dry lands represent a promising strategy to obtain proteins and peptides for feed. Human beings need to examine their eating habits and search for food products that do not put the sustainability of the environment and society at risk. 

## 7. Conclusions

In recent years, there has been an increasing interest in studying and researching arid and semiarid zones due to their extreme characteristics, such as prolonged periods of drought, sporadic rains with low average amounts, and generally high temperatures in the day and low temperatures at night. These conditions force some native species to adapt and carry out complex metabolic processes that originate compounds of interest, such as proteins and bioactive peptides, which can be used for human consumption. Generally, bioactive peptides and proteins are obtained by humans from conventional sources such as meat and dairy products. According to the Food and Agriculture Organization of the United Nations (FAO), the demand for protein of animal origin will continue to increase in future years. This will further increase the demand for food-grade protein. However, the disadvantages of this type of industry are known: its intensive production of dairy and slaughter cattle causes damage to the environment due to greenhouse gas emissions and the excessive use of water. The implementation of the production of these compounds through non-conventional sources such as insects, plants, and microorganisms is therefore of utmost importance for society and the environment. This is an interesting option since the population worldwide is growing, malnutrition is an increasing problem, and the 2030 agenda of the United Nations includes objectives for sustainable development, which include the fight against climate change and zero hunger.

## Figures and Tables

**Table 1:** Microorganisms isolated from arid zones with the capacity to produce protein compounds.

Enzyme	Source of Obtaining	Area	Reference
Protease	*Streptomyces* sp.	Saurashtra region, Gujarat, India	[35]
Protease	*Aspergillus flavus* and *Aspergillus niger*	Thiruvarur district, Tamilnadu	[36]
Hydrolase	*Diploschistes diacapsis* and *Lepraria crassissima*	Tabernas Desert, Spain	[37]
Hydrolase	*Glomus* sp., *Acaulospora* sp. and *Scutellospora* sp.	Baja California and Baja California Sur, México	[38]

**Table 2:** Plants from arid zones as a source of protein with functional properties.

Common Name/Part Used	Scientific Name	Country	Protein (%)	Molecules	Functional Properties	Source
Kashrangeeg	* Lablab purpureus * (L.)	Egypt	15	Not mentioned	Not mentioned	[45]
Hyacinth bean	* Lablab purpureus * (L.)	India	28	Glycosides viz. aloe-emodin, emodin, chrysophenol, rhein	Cytostatic potential and anticancer activities	[48]
Hyacinth bean	* Lablab purpureus * (L.)	South Africa	87.8	Arginine and lysine	Digestibility	[49]
Cacti pads	*Opuntia ficus-indica*	Mexico	7.7	Not mentioned	Not mentioned	[46]
Cladodes	*Opuntia ficus-indica*	Mexico	13.8	Not mentioned	Not mentioned	[50]
Mesquite	* Prosopis pallida *	Peru	9.5	Not mentioned	Not mentioned	[51]
Mesquite pods	* Prosopis laevigata *	Mexico	11.7	Not mentioned	Not mentioned	[52]
Sangri pods	*Prosopis cineraria*	India	31	Not mentioned	Antioxidant activity	[53]
Sangri pods	*Prosopis cineraria*	India	24.9	Not mentioned	Antifungal activity	[54]
Algarrobo	*Prosopis alba*	Argentina	85.5	Not mentioned	Anti-inflammatory and antioxidant activities	[55]
Flowers	*Calligonum azel*	Tunisia	17.8	Not mentioned	Not mentioned	[56]

## Data Availability

Similar articles published in MDPI Journals can be found at: https://www.mdpi.com/2304-8158/9/9/1303 and https://www.mdpi.com/2311-7524/9/12/1252.
